# Diagnostic Efficiency of Endometrial Sampling Methods and Risk Factors for Endometrial Carcinoma and Precursor Lesions in Premenopausal Women

**DOI:** 10.3390/jcm14113658

**Published:** 2025-05-23

**Authors:** Firdevs Öztürk, Saliha Sağnıç, Serap Fırtına Tuncer, Hasan Aykut Tuncer

**Affiliations:** 1 Department of Obstetrics and Gynecology, Akdeniz University, Antalya 07070, Turkey; firdevsustuner_7@hotmail.com; 2 Department of Obstetrics and Gynecology, Division of Gynecologic Oncology, Van Education & Research Hospital, Ministry of Health, Van 65300, Turkey; drsalihasagnic@hotmail.com; 3 Department of Obstetrics and Gynecology, Antalya Education & Research Hospital, Antalya 07100, Turkey; drserap.firtina@hotmail.com; 4 Department of Obstetrics and Gynecology, Division of Gynecologic Oncology, Akdeniz University, Antalya 07070, Turkey

**Keywords:** dilatation and curettage, endometrial carcinoma, endometrial hyperplasia, hysteroscopically directed biopsy, premenopause, Pipelle suction curettage

## Abstract

**Objective:** Accurate preoperative differentiation between benign endometrial conditions and malignancies is essential for guiding therapeutic interventions. However, high-quality evidence regarding the diagnostic accuracy of endometrial sampling techniques remains insufficient. This study aimed to evaluate the diagnostic efficiency of hysteroscopically directed biopsy, Pipelle suction curettage, and dilatation and curettage (D&C) for detecting endometrial hyperplasia or carcinoma in premenopausal women and to identify associated risk factors. **Methods:** A retrospective single-center cohort analysis was conducted on 2054 premenopausal women. Demographic, clinical, and obstetric data, along with biopsy techniques and histopathological findings, were recorded. Diagnostic accuracy of biopsy methods was compared against definitive surgical pathology. **Results:** The prevalence of endometrial hyperplasia and carcinoma was 5.6% and 1.0%, respectively. Hysteroscopically directed biopsy demonstrated superior diagnostic accuracy (AUC 0.957) compared to D&C (AUC 0.909) and Pipelle suction curettage (AUC 0.858). Sensitivity was highest for hysteroscopically directed biopsy (91.3%), followed by D&C (82.0%) and Pipelle suction curettage (71.7%), while specificity remained excellent across all methods (*p* < 0.001). Elevated BMI increased the risk of hyperplasia or carcinoma by 1.05 times per unit increase (OR = 1.054, *p* = 0.005), while hypertension nearly doubled the risk (OR = 1.99, *p* = 0.009). Multiparity showed protective effects, reducing risk with each additional delivery (OR = 0.877, *p* = 0.029). **Conclusions:** Hysteroscopically directed biopsy provides superior diagnostic accuracy for detecting endometrial hyperplasia and carcinoma in premenopausal women. Hypertension and elevated BMI increase risk, while multiparity offers protective benefits.

## 1. Introduction

Endometrial carcinoma is the sixth most common malignant disease, occurring mostly in postmenopausal women, but 5% to 30% of the disease is diagnosed in premenopausal women. According to the GLOBOCAN global cancer statistics, utilizing updated estimates from the International Agency for Research on Cancer (IARC), 420,242 new cases of corpus uteri carcinoma were reported worldwide in 2022 [1]. It is the most prevalent carcinoma of the female reproductive system in developed countries. Abnormal uterine bleeding (AUB) represents the predominant clinical manifestation in most premenopausal women with endometrial pathology [2]. Endometrial hyperplasia, principally induced by chronic unopposed estrogen exposure, represents the primary precursor lesion for endometrial carcinoma [3]. It carries a clinically significant risk of either concurrent endometrial carcinoma (up to 50%) [4,5,6,7] or progression to malignancy reported in 15% of cases over a median follow-up of 5.5 years, escalating to 28% over a median follow-up of 20 years [8,9].

The definitive diagnosis of benign endometrial pathologies, endometrial hyperplasia and carcinoma requires histopathological evaluation of endometrial tissue specimens acquired through biopsy, dilation and curettage (D&C), or hysterectomy. A comprehensive preoperative differentiation between these pathological entities is critical to guide appropriate therapeutic intervention. The potential coexistence of endometrial hyperplasia and carcinoma in women presenting with AUB necessitates the use of optimal endometrial sampling techniques for accurate histopathological diagnosis.

Traditionally, Pipelle suction curettage and D&C are considered effective in making a diagnosis, but inadequate sampling or the presence of cervical stenosis can lead to failure. Moreover, negative histology does not rule out the diagnosis of carcinoma. Non-visualized endometrial sampling has been reported to miss up to 62.5% of endometrial pathologies [10]. Hysteroscopy-guided endometrial sampling is increasingly replacing standard biopsy methods because it shows higher accuracy in detecting endometrial pathology [11]. However, its ability to correctly diagnose endometrial carcinoma and preinvasive lesions in premenopausal women with AUB before surgery is still not fully understood. The diagnostic accuracy of Pipelle suction curettage, D&C, and hysteroscopically directed biopsy was comparatively assessed in a limited patient cohort in retrospective studies and systematic reviews [12,13,14,15]. While several studies have demonstrated hysteroscopically guided biopsies to be diagnostically comparable or superior to both Pipelle suction curettage and D&C [16,17,18], other investigations have failed to replicate these findings [15,19,20,21], revealing ongoing controversy regarding optimal endometrial sampling methodology. Therefore, there is insufficient high-quality evidence regarding the diagnostic accuracy of endometrial sampling tests.

Several studies have assessed the diagnostic accuracy of endometrial sampling techniques in distinguishing between endometrial carcinoma and atypical hyperplasia within populations presenting with postmenopausal bleeding [11,22]. The analysis indicated that one technique exhibited superior diagnostic precision compared to the other in differentiating these conditions. However, these studies were limited by a small sample size and predominant inclusion of postmenopausal women; therefore, generalizing of these results to premenopausal population may be limited.

The accurate identification of high-risk patients with endometrial hyperplasia and carcinoma is critical for establishing an evidence-based foundation to guide both preventive interventions and clinical management strategies. While numerous reported risk factors may demonstrate causal relationships with endometrial hyperplasia and carcinoma, their effect estimates remain vulnerable to residual confounding and methodological biases—a well-documented limitation in epidemiological studies. A recent review examined 53 risk factors, among which only body mass index (BMI) and waist-to-hip ratio were strongly associated with increased endometrial carcinoma risk in premenopausal women. Parity was found to reduce the risk of disease. For the remaining identified risk factors, the evidence was inconclusive, so it is not yet possible to make definite conclusions [23].

Therefore, the primary aim of this study was to evaluate the diagnostic value of endometrial biopsy methods in premenopausal patients, and the secondary aim was to evaluate the risk factors for endometrial carcinoma or endometrial hyperplasia.

## 2. Materials and Methods

This retrospective single-center cohort analysis included 2054 premenopausal women who underwent endometrial biopsy at the Department of Gynecology and Obstetrics, Akdeniz University Faculty of Medicine between January 2015 and December 2020. Demographic characteristics (age, BMI), comorbidities, smoking, family history of endometrial carcinoma, obstetric history (gravidity, parity), imaging findings on transvaginal ultrasonography, endometrial sampling methods (hysteroscopically directed biopsy, Pipelle suction curettage, D&C), and histopathological findings were systematically collected from medical records. This study employed a consecutive case series design, with data analyzed from all 2054 eligible patients meeting inclusion criteria during the study period, without selective sampling. Exclusion criteria comprised (1) postmenopausal status, (2) pregnancy-related indications for endometrial sampling, (3) biopsies performed at external institutions, (4) history of gynecologic or breast malignancies (ovarian, endometrial, cervical, or breast carcinoma), (5) surveillance biopsies for non-bleeding indications, (6) patients with specific medication that affect endometrial status (e.g., tamoxifen/raloxifene, hormonal contraceptives, hormone replacement therapy, GnRH agonists/antagonist, medications for inducing ovulation (e.g., clomiphene citrate), progesterone antagonists, androgens and aromatase inhibitors, intrauterine devices, anticoagulants, NSAIDs, chemotherapy agents, antipsychotics (risperidone), immunosuppressants/corticosteroids), (7) patients affected by familiar diseases (i.e., HNPCC syndrome), (8) uterine metastases from cancer of other organs, and (9) patients with previous radiation therapy for another cancer in the pelvic area. This study was conducted in accordance with the ethical principles stated in the “Declaration of Helsinki” and approved by the institutional ethics committee (Akdeniz University Clinical Research Ethics Committee-2012-KAEK-20 70904504/565; date: 10 September 2020).

Endometrial sampling was performed using three clinical methods: (1) hysteroscopically directed biopsy, (2) Pipelle suction curettage, and (3) D&C. All endometrial sampling procedures were performed in strict accordance with standardized techniques described in current international guidelines [24,25,26,27]. Endometrial tissue specimens were immediately fixed in 10% neutral buffered formalin and transported to the institutional pathology department for standard histopathological processing and evaluation. All specimens underwent independent histopathological evaluation by multiple board-certified pathologists. In cases where the final pathology findings were discordant with the endometrial sampling results, the specimens were referred for secondary pathological review.

The highest grade of endometrial pathology, whether determined by endometrial biopsy or identified in a hysterectomy specimen, was designated as the definitive diagnosis and the highest-grade pathologies included endometrial carcinoma and endometrial hyperplasia. Receiver operating characteristic (ROC) curve analysis was performed to evaluate and compare the diagnostic accuracy of endometrial biopsy methods (hysteroscopically directed biopsy, Pipelle suction curettage, and D&C) against the reference standard. The area under the curve (AUC) was calculated for each technique, with 95% confidence intervals. For each biopsy method, diagnostic performance metrics were calculated at the optimal threshold: sensitivity, specificity, positive predictive value (PPV), and negative predictive value (NPV) with 95% confidence intervals. The value of *p* < 0.05 was considered statistically significant.

All statistical analyses were conducted using IBM SPSS Statistics (Version 26.0; IBM Corp., Armonk, NY, USA). Descriptive statistics were calculated according to data distribution characteristics: normally distributed continuous variables were summarized as mean ± standard deviation, while non-normally distributed variables were expressed as median (minimum-maximum). Categorical variables were reported as frequencies and percentages. Continuous variables were compared using independent Student’s *t*-tests for normally distributed data and Mann–Whitney U tests for non-normally distributed data, with distribution normality confirmed by Kolmogorov–Smirnov testing. Categorical variables were analyzed using Pearson’s chi-square test or Fisher’s exact test. The primary endpoint for analysis was significant endometrial pathology, defined as endometrial hyperplasia and endometrial carcinoma, confirmed by histopathological examination.

Univariate logistic regression analysis was conducted to evaluate potential predictors of significant endometrial pathology (hyperplasia or carcinoma) in premenopausal women, with all variables entered simultaneously using the Enter method. Variables demonstrating statistical significance (*p* < 0.05) in these initial analyses were subsequently included in the multivariable model (Backward LR) analysis. Since our study’s statistical analysis involved multiple variables, we used the Backward LR method to create a balanced model to identify independent variables by systematically excluding statistically insignificant predictors from the model, thereby enhancing the robustness and validity of the analytical framework. Model fit was assessed using three complementary measures: The Hosmer–Lemeshow goodness-of-fit test (*p* > 0.05 indicating adequate fit), Omnibus tests of model coefficients (χ^2^ with *p* < 0.001), and Nagelkerke’s pseudo r-square to estimate explained variance.

## 3. Results

The study cohort comprised 2054 premenopausal women with a mean age of 42.9 ± 6.3 years. The cohort had a mean BMI of 29.2 ± 5.9 kg/m^2^, with reproductive histories averaging 2.6 ± 1.7 pregnancies (range: 0–12) and 2.1 ± 1.2 deliveries (range: 0–12). The predominant clinical presentation was abnormal uterine bleeding (AUB), affecting 93% (*n* = 1910) of the study cohort. The majority of the patients (81.5% *n =* 1674) were non-smokers. The study cohort showed that 83% (*n =* 1712) of patients had no comorbidities. Isolated hypertension was present in 8% (*n =* 165), isolated diabetes mellitus in 5.5% (*n =* 112), and 3.2% (*n* = 65) had both conditions concurrently. Transvaginal ultrasonography revealed a median endometrial thickness of 12 mm (range 2–45). Uterine leiomyomas were identified in 26.3% (*n* = 540) of the study population by transvaginal ultrasonography.

Endometrial sampling was performed using three approaches, Pipelle suction curettage (47.3%, *n* = 971), hysteroscopically directed biopsy (30.6%, *n* = 628), and D&C (22.2%, *n* = 455), with method selection based on clinical indication and provider judgment. Histopathological evaluation revealed the following distribution of endometrial findings: endometrial polyps: 55.6% (*n* = 1141); proliferative/secretory endometrium: 37.0% (*n* = 760); endometrial hyperplasia: 5.6% (*n* = 116); endometrial carcinoma: 1.0% (*n* = 20); leiomyoma: 0.8% (*n* = 17). Surgical intervention was required for 557 patients (27.1% of the cohort), 75.8% (*n* = 422) of the patients underwent a hysterectomy, while 24.2% (*n* = 135) were treated with hysteroscopy. Histopathological evaluation of surgical specimens identified leiomyoma as the most common (42%) benign pathology. Notably, in 25.5% of cases, microscopic examination of endometrium showed benign endometrial patterns (proliferative, secretory, or normal). Endometrial polyps were identified in 17.6% of cases, whereas hyperplasia without atypia was detected in 3.9% of patients. Final histopathological diagnosis identified endometrioid intraepithelial neoplasia (EIN) in 6.8% (*n* = 38) and endometrial carcinoma in 4.1% (*n* = 23) cases.

ROC analysis was performed to compare the accuracy of the biopsy method to the definitive diagnostic method in the detection of endometrial hyperplasia and carcinoma (Table 1).

Among 60 patients with positive results for either of the two diagnostic categories based on the definitive diagnostic method, 43 cases were concordantly identified as positive via Pipelle suction curettage, yielding a sensitivity of 71.7%. Notably, all 975 patients with negative results across two diagnostic categories on the definitive diagnostic method were accurately classified as negative using the Pipelle suction curettage, demonstrating perfect specificity (100.0%). ROC curve analysis evaluating the diagnostic efficacy of D&C against the definitive diagnostic method demonstrated the following: Among 61 patients with positive results confirmed by the definitive diagnostic method, 50 cases were concordantly identified as positive via the D&C, yielding a sensitivity of 82.0%. Conversely, 409 out of 410 patients with negative results across two diagnostic categories based on the definitive diagnostic method were accurately classified as negative using D&C, demonstrating a specificity of 99.8%. Among 46 patients with positive results confirmed by definitive diagnostic method, 42 cases were concordantly identified as positive via hysteroscopy, yielding a sensitivity of 91.3%. Conversely, all 598 patients with negative results across two diagnostic categories based on definitive diagnostic method were accurately classified as negative using hysteroscopy, demonstrating specificity (100.0%).

**Figure 1:**
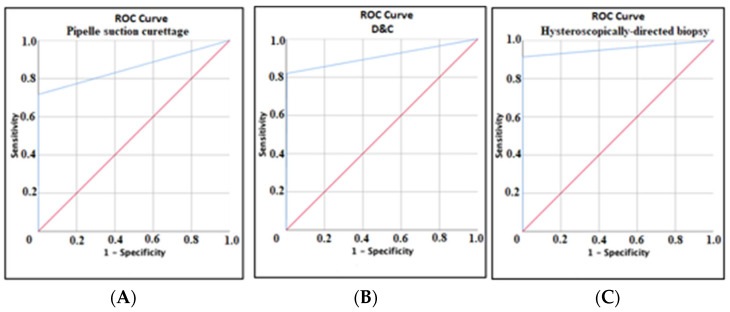
(**A**) ROC curve showing diagnostic efficacy of Pipelle suction curettage compared to definitive diagnostic method in the detection of endometrial hyperplasia and carcinoma. (**B**) ROC curve showing diagnostic efficacy of D&C compared to definitive diagnostic method in the detection of endometrial hyperplasia and carcinoma. (**C**) ROC curve showing diagnostic efficacy of hysteroscopically directed biopsy compared to definitive diagnostic method in the detection of endometrial hyperplasia and carcinoma (red arrows indicate the chance diagonal and blue arrows indicate the ROC curves of each sampling method).

Hysteroscopically directed biopsy showed superior diagnostic accuracy for detecting endometrial hyperplasia or carcinoma compared to both D&C (AUC 0.957 vs. 0.909) and Pipelle suction curettage sampling (AUC 0.957 vs. 0.858) (Table 1). The sensitivity was significantly higher for hysteroscopically directed biopsy (91.3%) than for D&C (82.0%) and Pipelle suction curettage (71.7%), while all methods maintained excellent specificity (hysteroscopically directed biopsy 100%; D&C: 99.8%; Pipelle suction curettage: 100%; all comparisons *p* < 0.001). Pipelle suction curettage demonstrated perfect positive prediction (PPV = 98.3%) and high negative predictive value (NPV = 100%) for detecting endometrial hyperplasia and carcinoma (Figure 1A). D&C demonstrated perfect predictive accuracy, with both positive and negative predictive values reaching 98% and 97.4%, respectively, detecting endometrial hyperplasia and carcinoma (Figure 1B). Hysteroscopically directed biopsy demonstrated excellent predictive accuracy as PPV was 100% and NPV was 99.3% (95% CI: 90–100) for detecting endometrial hyperplasia and carcinoma (Figure 1C). Hysteroscopically directed biopsy had the highest diagnostic efficiency.

Baseline demographic, clinical, and obstetric characteristics of patients with and without histologically confirmed endometrial hyperplasia and carcinoma were compared (Table 2).

Patients with endometrial hyperplasia or carcinoma demonstrated significantly higher BMI values (mean 31.0 ± 0.8 kg/m^2^) compared to those without these pathologies (mean 28.9 ± 5.7 kg/m^2^; *p* = 0.002). Women with endometrial hyperplasia or carcinoma exhibited significantly lower gravidity (mean 2.3 ± 1.7 vs. 2.6 ± 1.7; *p* = 0.002) and parity (mean 1.8 ± 1.1 vs. 2.1 ± 1.3; *p* = 0.007) compared to those without these pathologies. The prevalence of systemic comorbidities was significantly higher in patients with endometrial hyperplasia or carcinoma compared to those without these pathologies (22.8% vs. 16.2%, *p* = 0.021).

Univariate logistic regression analysis revealed that each one kg/m^2^ increase in body mass index (BMI) was associated with 1.054 times risk for endometrial hyperplasia or carcinoma (OR = 1.054, 95% CI: 1.016–1.094, *p* = 0.005) among premenopausal women. Higher gravidity (OR = 0.877, 95% CI: 0.780–0.986, *p* = 0.029) and parity (OR = 0.840, 95% CI: 0.720–0.980, *p* = 0.027) demonstrated significant protective effects against endometrial hyperplasia and carcinoma. Hypertensive women demonstrated nearly two-fold greater odds of endometrial hyperplasia or carcinoma compared to normotensive controls (adjusted OR = 1.99, 95% CI: 1.19–3.34, *p* = 0.009). All variables demonstrating significance (*p* < 0.05) in univariate analysis were entered into the multivariable logistic regression model. Multivariable analysis demonstrated that each 1 kg/m^2^ increase in BMI was associated with a 1.05 times risk for endometrial hyperplasia or carcinoma (OR = 1.053, 95% CI: 1.012–1.096, *p* = 0.011). Hypertension conferred 2.3-fold greater odds of these endometrial pathologies compared to normotensive women (adjusted OR = 2.29, 95% CI: 1.17–4.50, *p* = 0.016) (Table 3).

## 4. Discussion

The primary aim of this study was to determine the diagnostic efficiency of hysteroscopically directed biopsy, Pipelle suction curettage, and D&C, and secondarily to identify risk factors for endometrial hyperplasia and carcinoma in premenopausal patients. Our analysis identified hysteroscopically directed biopsy as the most accurate diagnostic modality for detecting endometrial hyperplasia and carcinoma in premenopausal women, demonstrating a sensitivity of 91.3% and specificity of 100%. This conclusion is supported by its superior diagnostic performance, as evidenced by the highest AUC value among the evaluated methods. AUC quantifies the overall diagnostic accuracy of a test by measuring its ability to discriminate between the diseased and non-diseased groups. The closer the AUC value is to 1, the more accurate the diagnostic test becomes. In the present study, the AUC of hysteroscopically directed biopsy was calculated as 0.957, showing excellent accuracy. The AUC score for Pipelle suction curettage was 0.858, showing that it effectively distinguishes between cases with high accuracy.

Although endometrial carcinoma predominantly affects postmenopausal women, it warrants inclusion in the differential diagnosis of premenopausal patients presenting with abnormal uterine bleeding or risk factors. Epidemiological studies estimate that 5–30% of endometrial carcinoma cases are diagnosed among premenopausal women. Nevertheless, robust evidence from meta-analyses indicates that this population exhibits a low prevalence of endometrial carcinoma or atypical endometrial hyperplasia [28]. Physiological endometrial variations, characterized by secretory and proliferative phases, and benign endometrial pathologies such as endometrial polyps are the most frequently identified entities in premenopausal women [29,30]. This distribution aligns closely with the histopathological patterns identified in the present study, where the incidence of endometrial hyperplasia and carcinoma was lower than those reported in the prior literature. This discrepancy may be attributed to the fact that nearly all participants were symptomatic, as premenopausal women presenting with abnormal uterine bleeding are predominantly associated with benign endometrial pathologies (e.g., polyps, hormonal imbalances) rather than malignant or premalignant conditions. The higher prevalence of benign etiologies in this demographic likely contributed to the reduced detection rates of hyperplasia and carcinoma compared to population-based studies, which often include asymptomatic or high-risk cohorts.

Current clinical guidelines categorize premenopausal women under 45 years of age presenting with AUB as a low-risk demographic for endometrial carcinoma. Consequently, routine endometrial biopsy in this population is often deemed unwarranted. The American College of Obstetricians and Gynecologists (ACOG) recommends office-based endometrial biopsy for premenopausal patients with elevated risk factors for endometrial hyperplasia or neoplasia. If insufficient tissue is obtained through standard office sampling methods, hysteroscopy should be performed [31]. Based on the findings of our study, we may suggest prioritizing hysteroscopically directed biopsy over standard office endometrial sampling methods in premenopausal women presenting with AUB and risk factors for endometrial hyperplasia or carcinoma.

Histopathological evaluation via endometrial biopsy or cytological sampling has been demonstrated to significantly reduce disease-related morbidity and mortality rates [32]. Pipelle suction curettage offers a cost-effective alternative to traditional D&C by obviating the need for general anesthesia, thereby reducing both procedural complexity and healthcare expenditures. However, the diagnostic evaluation of endometrial specimens obtained via this method may be complicated by histopathological overlap, insufficient specimen acquisition, or diagnostic discordance among pathologists. Consequently, obtaining sufficient tissue volume during biopsy procedures is imperative to ensure diagnostic accuracy and mitigate the risk of false-negative results in clinical practice. Pipelle suction curettage and D&C, as non-visualized endometrial sampling techniques, are inherently constrained by their inability to provide direct visualization of the endometrial cavity during tissue acquisition. This methodological limitation aligns with prior research demonstrating that outpatient endometrial biopsy modalities exhibit suboptimal sensitivity and lack predictive reliability in diagnosing endometrial carcinoma, particularly for detecting focal lesions or early-stage malignancies [21,24,33,34,35]. In a retrospective cohort analysis, Piatek et al. evaluated the diagnostic adequacy of endometrial biopsy techniques, specifically comparing Pipelle suction curettage and D&C. Their findings indicated that neither method reliably ensures sufficient specimen acquisition [14]. A cross-sectional study conducted by Utida et al. to evaluate the diagnostic concordance between Pipelle suction curettage and hysteroscopy-guided biopsy demonstrated perfect agreement (100%) between the two modalities in detecting endometrial carcinoma. However, the accuracy of Pipelle suction curettage was significantly lower for diagnosing endometrial polyps, with notable discrepancies observed in histopathological correlation [17]. These findings, while promising for carcinoma detection, warrant cautious interpretation due to the study’s limited statistical power secondary to its small cohort size.

While a systematic review of retrospective studies has demonstrated that Pipelle suction curettage exhibits superior diagnostic performance compared to alternative endometrial sampling modalities in detecting endometrial carcinoma and endometrial hyperplasia [21], hysteroscopy-guided biopsy remains the gold-standard approach for targeted biopsy, achieving the highest diagnostic accuracy in histopathological evaluation when integrated with curettage [24,34,36,37]. It enables direct visualization of the uterine cavity, facilitating immediate histopathological diagnosis and targeted therapeutic intervention. This heightened diagnostic accuracy persists across diverse patient populations, irrespective of age or menopausal status [24,38,39,40]. This approach is further supported by robust evidence demonstrating its efficacy in reducing sampling errors and false-negative rates, thereby optimizing clinical outcomes in patients with suspected endometrial neoplasia or hyperplasia [11,12,41,42]. Therefore, in patients diagnosed with atypical endometrial hyperplasia via outpatient endometrial biopsy, preoperative confirmation of the diagnosis via hysteroscopy should be attempted to avoid overlooking high-grade malignancies and to prevent missing the opportunity for sentinel lymph node dissection [11]. Contrary to this assertion, emerging evidence from a recent comparative study revealed moderate concordance across three endometrial sampling modalities (Pipelle suction curettage, dilation and curettage, and hysteroscopy-guided biopsy) when correlated with final hysterectomy specimen histology. Notably, the authors advocated for the preferential use of Pipelle suction curettage as a diagnostically adequate and clinically viable method for endometrial carcinoma evaluation, citing its comparable diagnostic yield to hysteroscopy-guided biopsy, procedural simplicity, and cost-effectiveness in resource-constrained settings [16]. However, the study population was not randomized to a diagnostic method, and the D&C group exhibited a smaller sample size relative to the Pipelle suction curettage and hysteroscopy-guided biopsy cohorts.

While hysteroscopy-directed biopsies serves as a critical diagnostic modality for identifying endometrial hyperplasia and carcinoma, its utility in determining endometrial tumor histotype and histological grade remains a subject of debate, with existing studies yielding conflicting results [11,43,44,45]. For instance, Phelippeau et al. reported that both Pipelle suction curettage and operative hysteroscopy exhibit limited reliability in definitively assessing histological subtype and tumor grade, underscoring discrepancies between preoperative and postoperative histopathological findings [44]. Conversely, Su et al. demonstrated that hysteroscopy provides superior diagnostic accuracy for tumor grading compared to D&C, with significantly lower discordance rates (2.6% vs. 12.6%) between preoperative and final surgical pathology [45]. Each diagnostic method has a potential risk of underestimation or overestimation of tumor grade in endometrial carcinoma, as well as misclassification of histologic subtypes (endometrioid versus non-endometrioid), which may compromise diagnostic accuracy. The optimal diagnostic approach for preoperative evaluation of endometrial carcinoma—particularly in reliably determining tumor grade and histopathological classification remains uncertain. Large-scale prospective, randomized controlled trials are required to establish the most accurate diagnostic method.

The aforementioned studies encompass heterogeneous cohorts comprising both premenopausal and postmenopausal patients. In contrast, our investigation exclusively focuses on premenopausal individuals, representing a narrower and more homogeneous larger cohort compared to prior population-based studies. We specifically evaluated the efficacy of these three diagnostic modalities in premenopausal patients, as this demographic presents unique clinical challenges. Given their status within the reproductive age group, physiological endometrial changes associated with the menstrual cycle—such as cyclical proliferation and shedding—may confound the differentiation between pathological and benign findings during hysteroscopic evaluation. Consequently, our study’s targeted approach offers a distinct advantage in refining diagnostic accuracy and enabling precise, individualized therapeutic planning for this population.

We identified BMI and hypertension as independent predictors of endometrial hyperplasia and carcinoma in premenopausal women. A one-unit increase in BMI was associated with a 1.05-fold increased likelihood of endometrial hyperplasia and carcinoma, while hypertension was associated with a 2.3-fold higher risk. Excess body weight has higher risk of carcinoma in multiple anatomical locations including the endometrium. The rising prevalence of obesity correlates with an increased incidence of newly diagnosed endometrial carcinoma cases [36]. In obese women, chronic endogenous estrogen exposure, driven by excess adiposity, serves as a primary etiological factor in the pathogenesis of endometrial hyperplasia and carcinoma. This relationship arises due to the strong correlation between adipose tissue accumulation and elevated estradiol levels [3]. Numerous studies and large-scale meta-analyses have robustly established a significant association between obesity and endometrial carcinoma [23,46,47]. Furthermore, meta-analyses have demonstrated a strong association between hypertension and endometrial carcinoma, although the underlying biological mechanisms remain incompletely elucidated [47]. Our findings corroborate the existing literature, demonstrating that obesity and hypertension significantly increase the risk of endometrial hyperplasia and carcinoma.

The assessment of premenopausal women presenting with abnormal uterine bleeding constitutes a clinical indication for endometrial biopsy, particularly in individuals with underlying risk factors such as nulliparity [14,33,34]. Consistent with our findings, epidemiological studies have demonstrated that multiparous women exhibit a decreased risk of endometrial carcinoma development compared to nulliparous individuals. Risk reduction with parity may be attributed to pregnancy-induced hormonal alterations, which are predominantly characterized by a pronounced shift toward progesterone dominance. This physiological transition exerts anti-proliferative effects on the endometrium, thereby mitigating the risk of malignant transformation [23]. Moreover, chronic anovulation induces a state of unopposed estrogen exposure due to the absence of progesterone’s counteractive effects, thereby promoting sustained endometrial proliferation. This pathophysiological mechanism elevates the risk of developing endometrial hyperplasia and, ultimately, endometrial carcinoma. Numerous epidemiological investigations have demonstrated that parity has been shown to be inversely associated with the risk of endometrial pathologies, further corroborating its protective role in this clinical context [23,46,48,49,50].

The primary limitations of this study include its retrospective single-center design, which inherently introduces the potential for selection bias, incomplete data collection (risk factors for endometrial carcinoma such as lifestyle factors (physical activity levels, sedentary behavior), metabolic parameters (presence of metabolic syndrome, insulin resistance), daily dietary patterns, anthropometric indices (waist-to-hip ratio, waist circumference, weight gain, weight, hip circumference, and height), reproductive variables (age at menarche, age at last birth, breastfeeding duration, and history of fertility treatment), and restricted generalizability of findings. Another limitation of our study is that we did not include recently discovered technique called liquid-based endometrial cytology for investigating tumor cells in body fluids, which may be a promising method for the detection of endometrial carcinoma. A further limitation of this study is the inability to conduct a comparative analysis of various mechanical hysteroscopic biopsy techniques (e.g., tissue retrieval systems, 5Fr forceps, resectoscope-directed deep endometrial sampling), as procedural documentation within institutional medical records lacked granular details regarding instrumentation. An additional limitation of this study arises from the omission of tumor grade documentation in the pathology reports of patients diagnosed with endometrial carcinoma. Consequently, we were unable to perform a comparative analysis between the histopathological characteristics of tumors identified via preoperative endometrial sampling modalities (e.g., Pipelle suction curettage, hysteroscopy) and those reported in the final surgical pathology specimens.

## 5. Conclusions

In conclusion, hysteroscopy-directed biopsy exhibits superior diagnostic accuracy and higher sensitivity compared to D&C and Pipelle suction curettage in detecting endometrial hyperplasia and carcinoma among premenopausal populations. Therefore, it may replace D&C and Pipelle suction curettage as the preferred diagnostic modality. All three modalities demonstrated comparable specificity. Hypertensive premenopausal patients with higher BMI are at significantly higher risk for endometrial hyperplasia and carcinoma. Conversely, increased parity and gravidity exhibit a protective association against significant endometrial pathology.

## Figures and Tables

**Table 1:** ROC analysis showing the diagnostic efficiency of Pipelle suction curettage, D&C, and hysteroscopic biopsies compared to definitive diagnostic method in the detection of endometrial hyperplasia and endometrial carcinoma.

	AUC	%95 CI	Sensitivity	Specificity	PPV	NPV	*p*
Pipelle suction curettage	0.858	0.789–0.928	71.7	100.0	98.3	100.0	<0.001
D&C	0.909	0.851–0.966	82.0	99.8	98.0	97.4	<0.001
Hysteroscopically- directed biopsy	0.957	0.909–1.000	91.3	100.0	100.0	99.3	<0.001

Note: AUC: Area under the curve. CI: confidence interval. PPV: positive predictive value. NPV: negative predictive value. D&C: dilatation and curettage.

**Table 2:** Comparison of baseline demographic, clinical, and obstetric characteristics of patients with and without endometrial hyperplasia and carcinoma.

	Endometrial Hyperplasia and Carcinoma (−) (*n* = 1918)	Endometrial Hyperplasia and Carcinoma (+) (*n* = 136)	*p*
Age (years), mean ± SD	42.89 ± 6.22	43.65 ± 6.19	0.739 *
BMI (kg/m^2^), mean ± SD	28.92 ± 5.70	30.98 ± 0.7	0.002 **
Gravida, mean ± SD	2.61 ± 1.66	2.29 ± 1.70	0.002 **
Parity, mean ± SD	2.07 ± 1.25	1.83 ± 1.13	0.007 **
Endometrial thickness (mm) (Min-Max)	12 (2–45)	12 (5–38)	0.234 **
Smoking, *n* (%)			0.971 ***
No	1563 (81.5)	111 (81.6)	
Yes	355 (18.5)	25 (18.4)	
Comorbidity, *n* (%)			0.021 ***
None	1607 (83.8)	105 (77.2)	
HT	146 (7.6)	19 (14.0)	
DM	117 (5.6)	5 (3.7)	
HT + DM	58 (3.0)	7 (5.1)	
Symptom, *n* (%)			0.233 ***
Asymptomatic	87 (4.5)	2 (1.5)	
Abnormal uterine bleeding	1781 (92.9)	130 (95.6)	
Pelvic pain	50 (2.6)	4 (2.9)	
Endometrial fluid, *n* (%)			0.168 ****
No	1182 (98.1)	136 (100.0)	
Yes	36 (1.9)	0 (0.0)	

Note: BMI: body mass index; HT: hypertension; DM: diabetes mellitus; * *t* test, ** Mann–Whitney U test; *** Pearson chi-square test; **** Fisher’s exact test.

**Table 3:** Determination of risk factors in premenopausal patients by logistic regression analysis.

Univariate Logistic Regression	B	SE	Exp (B)	%95 GA	*p*
BMI	0.05	0.19	1.054	1.016–1.094	0.005 *
Gravida	−0.131	0.060	0.877	0.780–0.986	0.029 *
Parity	−0.174	0.079	0.840	0.720–0.980	0.027 *
Comorbidity					
None					0.025 *
HT	0.689	0.264	1.992	1.187–3.341	0.009 *
DM	−0.335	0.468	0.715	0.286–1.791	0.474 *
HT + DM	0.614	0.413	1.847	0.823–4.147	0.137 *
**Multivariate Logistic** **Regression**	**B**	**SE**	**Exp (B)**	**%95 GA**	***p***
BMI	0.052	0.020	1.053	1.012–1.096	0.011 *
Comorbidity					
None					0.085 *
HT	0.829	0.344	2.291	1.168–4.495	0.016 *
DM	0.034	0.513	1.035	0.378–2.830	0.947 *
HT + DM	0.638	0.523	1.892	0.680-5.269	0.212 *

Note: BMI: body mass index; HT: hypertension; DM: diabetes mellitus; * logistic regression analysis (Enter); Omnibus test of model coefficient = 0.000; Hosmer–Lemeshow test = 0.241; Nagelkerke R square = 0.095.

## Data Availability

The datasets used or analyzed during the current study are available from the corresponding author H.A.T. on reasonable request.

## Abbreviations

The following abbreviations are used in this manuscript:

ACOG	American College of Obstetricians and Gynecologists
AUB	abnormal uterine bleeding
AUC	area under the curve
BMI	body mass index
DM	diabetes mellitus
D&C	dilatation and curettage
EIN	endometrioid intraepithelial neoplasia
HT	hypertension
IARC	International Agency for Research on Cancer
NPV	negative predictive value
OR	odds ratio
PPV	positive predictive value
ROC	receiver operating characteristic
